# 2B4 Is Dispensable for T-Dependent B Cell Immune Responses, but Its Deficiency Leads to Enhanced T-Independent Responses Due to an Increase in Peritoneal Cavity B1b Cells

**DOI:** 10.1371/journal.pone.0137314

**Published:** 2015-08-31

**Authors:** Avijit Ray, Cheng-Yin Yuan, Nichole M. Miller, Hong Mei, Bonnie N. Dittel

**Affiliations:** 1 Blood Research Institute, BloodCenter of Wisconsin, Milwaukee, Wisconsin, United States of America; 2 Department of Microbiology and Molecular Genetics, Medical College of Wisconsin, Milwaukee, Wisconsin, United States of America; Institut National de la Santé et de la Recherche Médicale (INSERM), FRANCE

## Abstract

The signaling lymphocyte activation molecule (SLAM) family plays important roles in adaptive immune responses. Herein, we evaluated whether the SLAM family member 2B4 (CD244) plays a role in immune cell development, homeostasis and antibody responses. We found that the splenic cellularity in *Cd244* ^*-/-*^ mice was significantly reduced due to a reduction in both CD4 T cells and follicular (Fo) B cells; whereas, the number of peritoneal cavity B cells was increased. These findings led us to examine whether 2B4 modulates B cell immune responses. When we examined T-dependent B cell responses, while there was no difference in the kinetics or magnitude of the antigen-specific IgM and IgG_1_ antibody response there was a reduction in bone marrow (BM) memory, but not plasma cells in *Cd244* ^*-/-*^ mice. When we evaluated T-independent immune responses, we found that antigen-specific IgM and IgG_3_ were elevated in the serum following immunization. These data indicate that 2B4 dampens T-independent B cell responses due to a reduction in peritoneal cavity B cells, but has minimal impact on T-dependent B cell responses.

## Introduction

2B4 is a member of the signaling lymphocyte activation molecule (SLAM)-related receptor family and is also known as SLAMF4 and CD244 [1]. All members of the SLAM family share a similar structure, including an extracellular domain, a transmembrane region, and a tyrosine rich cytoplasmic region [1]. Unlike most SLAM family members, 2B4 does not bind via hemophilic interactions, but binds to CD48, which is broadly expressed by hematopoietic cells and functions as an adhesion and co-stimulatory receptor for both B and T cells [2]. By means of their immunoreceptor tyrosine-based switch motifs (ITSM) in the cytoplasmic domain, SLAM family receptors signal by interacting with members of the SLAM-associated protein (SAP) (SH2D1A) family of adaptors [1]. The SAP adaptors couple SLAM proteins to biochemical signaling pathways mediating the various biological functions of the SLAM family [1, 3].

2B4 expression by B cells has been best studied in humans where its expression by all B cell subsets was reported to be very low to absent as compared to other SLAM family members [4]. However, upon transformation with Epstein-Barr virus, 2B4 expression was induced with up to 79% of blasts staining positive [5]. 2B4 expression was also upregulated by pokeweed mitogen with 5–38% of B cell blasts positive [5]. Interactions between CD48 and 2B4 can lead to signaling through both receptors [2, 6]. CD48 signaling in B cells leads to homotypic adhesion, proliferation and/or differentiation, release of inflammatory effector molecules and isotype class switching [2, 7, 8]. In addition, all of these processes are also elicited in T cells via CD48 ligation with the addition of promoting their activation and/or cytotoxicity [2]. 2B4 signaling requires SAP or EWS-activated transcript 2 (EAT-2; also called SH2D1B) [6, 9–11]. In CD8 T cells and NK cells 2B4 has been reported to exert both positive and negative regulation [9–11]. A specific role for 2B4 in B cells has not been reported.

Here we investigated the role of 2B4 in B cells and found that *Cd244* ^*-/-*^ mice have a significant reduction in splenic cellularity that was due to a reduction in CD4 T and follicular (Fo) B cells. We also found that peritoneal cavity B cells were increased in *Cd244* ^*-/-*^ mice due to a significant increase in B1b and B2, but not B1a cells. When we examined 2B4 expression, we found that B cell subsets expressed no to very low levels of 2B4. Following a T-dependent immune response, there was no difference in the kinetics and the magnitude of the antigen-specific IgM and IgG_1_ response between WT and *Cd244* ^*-/-*^ mice. However, late in the response there was a significant decrease in the number of bone marrow (BM) memory B cells in *Cd244* ^*-/-*^ mice. Following immunization with a T-independent antigen, *Cd244* ^*-/-*^ mice exhibited a significant increase in antigen-specific IgM production on day 14 and isotype-class switched IgG_3_ on days seven and 14. These data indicate that even though a global deficiency in 2B4 is associated with reduced numbers of Fo and BM memory B cells it has minimal impact on T-dependent B cell responses. In contrast, the increase in peritoneal cavity B cells in *Cd244* ^*-/-*^ mice is directly correlated to an increase in the T-independent immune response.

## Materials and Methods

### Ethics statement

All animal protocols used were approved by the Medical College of Wisconsin’s Institutional Animal Care and Use Committee. We monitored immunized animals for adverse health issues and used appropriate methods of euthanasia including isoflurane or CO_2_ followed by cervical dislocation.

### Mice and reagents

C57BL/6 (WT) mice were purchased from The Jackson Laboratories (Bar Harbor, ME).

*Cd244* ^-/-^ mice on the C57BL/6 background [12], were kindly provided by Dr. Raymond Welsh (University of Massachusetts Medical Center, Worcester, MA). Mice were maintained in the Translational Biomedical Research Center of the Medical College of Wisconsin and both genders were used for experiments in an age (6–12 week) and gender-matched manner. Anti-CD45R-PE-Texas Red, anti-CD45R-PE, anti-GL7-FITC and anti-CD5-APC were purchased from BD Biosciences (San Jose, CA). CD21-eFluor 450, anti-CD23-PE-Cy7, anti-CD93-biotin, anti-CD4-APC-eFluor 780, anti-CD8-PE-Cy7, anti-TCRβ-FITC, anti-TCRβ-PE, anti-CD11b-Alexa Fluor 488, anti-CD244, anti-Foxp3-PE, anti-CD19-Alexa 700, anti-CD4-PE, anti-IgG-FITC and Streptavidin PE-Cy5.5 were purchased from eBioscience (San Diego, CA). Anti-CD11b-Brilliant Violet 605, anti-CD11c-PE, anti-NK1.1-APC, anti-IgM-APC Cy7, anti-IgD-Pacific Blue, anti-CD38-Alexa Fluor 647, anti-CD138-APC and rat anti-mouse IgG_2a_-biotin were purchased from Biolegend (San Diego, CA). Anti-IgM-FITC and the SBA Clonotyping System-B6/C57J-HRP were purchased from Southern Biotech (Birmingham, AL). 4-Hydroxy-3-nitrophenylacetyl (NP)-ficoll, NP-Chicken Gamma Globulin (CGG), NP(24)-PE and NP-BSA were purchased from Biosearch Technologies (Novato, CA).

### Cell isolation and flow cytometry

Single cell suspensions were prepared from spleens and 1 x 10^6^ cells were stained with specific antibodies. Peritoneal cavity B cells were isolated as described [13]. For 2B4 expression, anti-CD244 staining was followed by anti-IgG_2a_-biotin and then Strepavidin-PE-Cy5.5 to amplify the signal. Samples were acquired with a LSR II flow cytometer (Becton Dickinson, San Jose, CA) and data were analyzed using FlowJo software (Tree Star, Ashland, OR).

### Immunization and serum collection

For T-independent humoral responses, WT and *Cd244* ^-/-^ mice were i.p. immunized with 30 μg NP-ficoll, as described [14]. Serum was collected 0, 7 and 14 days after immunization. For T-dependent humoral responses, 8–12 week old WT and *Cd244* ^-/-^ mice were immunized with 30 μg alum-precipitated NP-CGG with a NP to CGG ratio of 27:1 via i.p injection, as described [15]. Serum was collected 0, 7, 10, 14, 28 and 42 days after immunization.

### ELISA

To determine antigen-specific serum IgM, IgG_1_ and IgG_3_ titers, 96 well flat-bottom Nunc MaxiSorp Microwell plates were coated overnight with 50 μg /ml NP_25_-BSA (Biosearch Technologies, Novato, CA) diluted in carbonate buffer. The plates were blocked with 1% BSA for 2 h at room temperature prior to the addition of serially diluted serum samples. The serum samples were diluted with 1% BSA and two-fold dilutions were performed: 1/800 to 1/6400 for IgM, 1/10000 to 1/80000 for IgG_1_, and 1/200 to 1/3200 for IgG_3_. Subsequently, HRP-conjugated anti-IgM, anti-IgG_1_ or anti-IgG_3_ were added to the wells and NP-specific Ig levels were detected using 2,2'-Azinobis (3-ethylbenzothiazoline-6-sulfonic acid)-diammonium salt (ABTS) substrate (Southern Biotechnology, Birmingham, AL). Absorbance was measured at 405 nm.

### Statistical analysis

WT and *Cd244* ^-/-^ groups were compared using a two-tailed unpaired t-test. A *p* value < 0.05 was considered significant.

## Results and Discussion

### *Cd244* ^*-/-*^ mice have reduced numbers of CD4 T cells and Fo B cells

**Fig 1:**
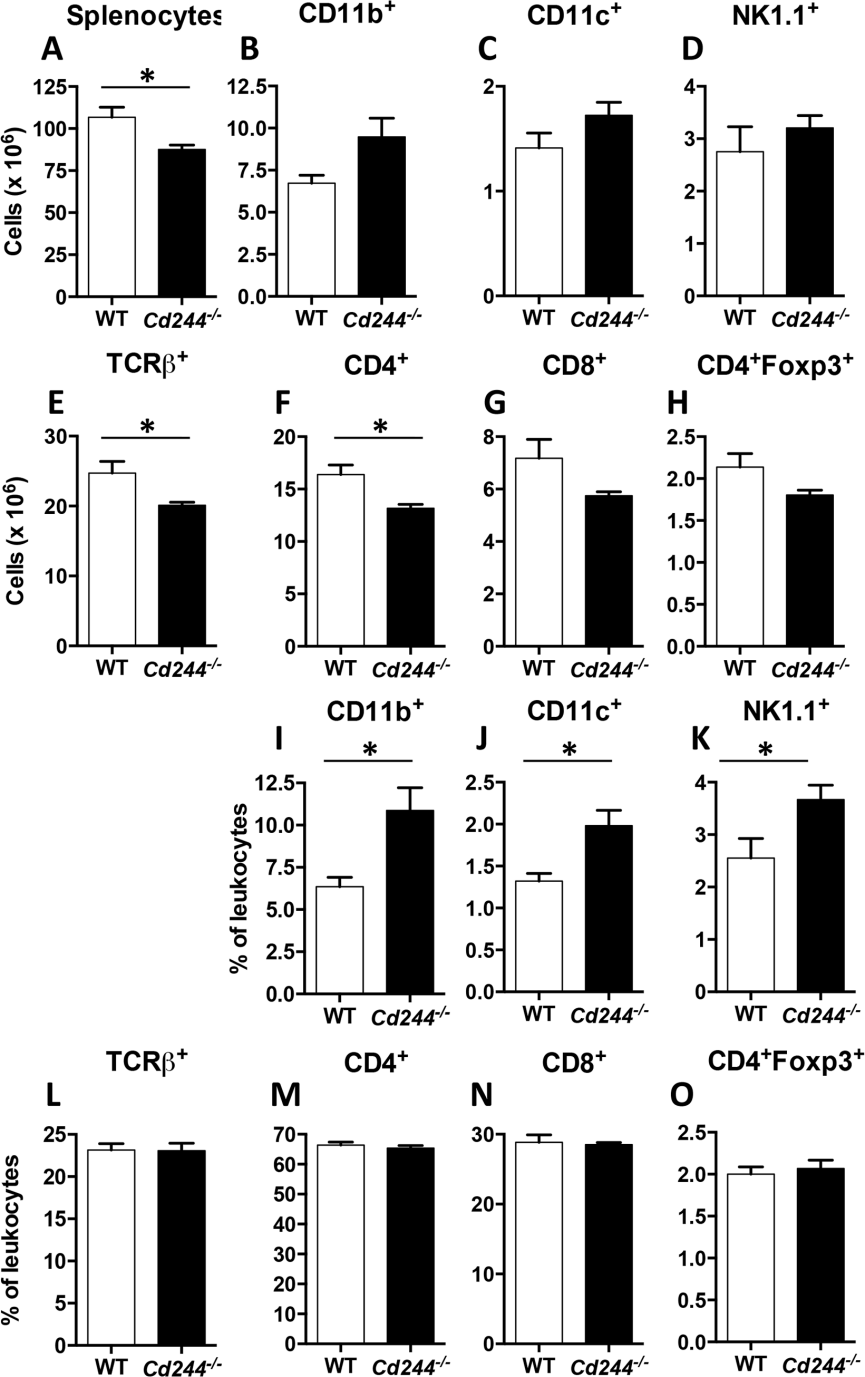
The cellularity and absolute number of CD4 T cells is reduced in the spleen of *Cd244* ^*-/-*^ mice. The absolute number of mononuclear (A), CD11b^+^ (B), CD11c^+^ (C), NK1.1^+^ (D), TCRβ^+^ (E), TCRβ^+^CD4^+^ (F), TCRβ^+^CD8^+^ (G) and CD4^+^Foxp3^+^ (H) cells in the spleen of WT and *Cd244* ^*-/-*^ mice was determined by flow cytometry. The percentage of CD11b^+^ (I), CD11c^+^ (J), NK1.1^+^ (K), TCRβ^+^ (L), TCRβ^+^CD4^+^ (M), TCRβ^+^CD8^+^ (N) and CD4^+^Foxp3^+^ (O) cells in the spleen of WT and *Cd244* ^*-/-*^ mice was determined by flow cytometry. Data are shown as the mean ± SE of four mice. *p<0.05.

In our mouse colony, we found that *Cd244* ^*-/-*^ mice had a significant reduction in splenic cellularity (Fig 1A). This finding was not reported in the original study describing the *Cd244* ^*-/-*^ mice [12]. In this same study, it was reported that a global deficiency in 2B4 did not alter the frequency (percentage) of CD3^+^, CD4^+^, CD8^+^, CD11b^+^, CD19^+^ nor NK1.1^+^ cells in the spleen [12]. However, the percentage does not reflect changes in absolute cell number. Thus, we repeated the splenic phenotyping and calculated absolute numbers of cells to determine the specific immune cell populations that are reduced in *Cd244* ^*-/-*^ mice in our colony. We found no difference in the absolute number of CD11b^+^ myeloid cells (Fig 1B), CD11c^+^ dendritic cells (Fig 1C) or NK1.1^+^ NK cells (Fig 1D) between WT and *Cd244* ^*-/-*^ mice. In contrast, there was a significant reduction in αβ T cells in the spleen (Fig 1E). Further analysis revealed that the reduction was due to lower numbers of CD4^+^ T cells (Fig 1F). In addition, while a trend for lower numbers of CD8^+^ T cells (p = 0.1) (Fig 1G) and CD4^+^Foxp3^+^ T regulatory cells (p = 0.11) (Fig 1H) was observed in *Cd244* ^*-/-*^ mice, it did not reach significance. When we examined the percentage of the above immune cell populations in the spleen, the non-significant increase in CD11b^+^ myeloid cells (p = 0.06) was reflected in a significant increase in percentage of total CD11b^+^ cells (Fig 1I) and percentage of the CD11b^+^ dendritic (Fig 1J) and NK cells (Fig 1K) subsets. Interestingly, even though significance was observed for absolute number there was no difference in the percentage of αβ T cells (Fig 1L) or CD4 T cells (Fig 1M). In addition, there was no difference in the percentage of CD8 T cells (Fig 1N) or in Treg (Fig 1O). It is not clear why we observed an increase in the percentage of myeloid cells that was not seen in the original report describing *Cd244* ^*-/-*^ mice, but likely reflects differences in composition of the microbiota in the two animal colonies. Nevertheless, our findings that significant differences in percentages is not reflected by the absolute cell number highlights why only analyzing changes in the percentages of specific cell subsets can be misleading.

**Fig 2:**
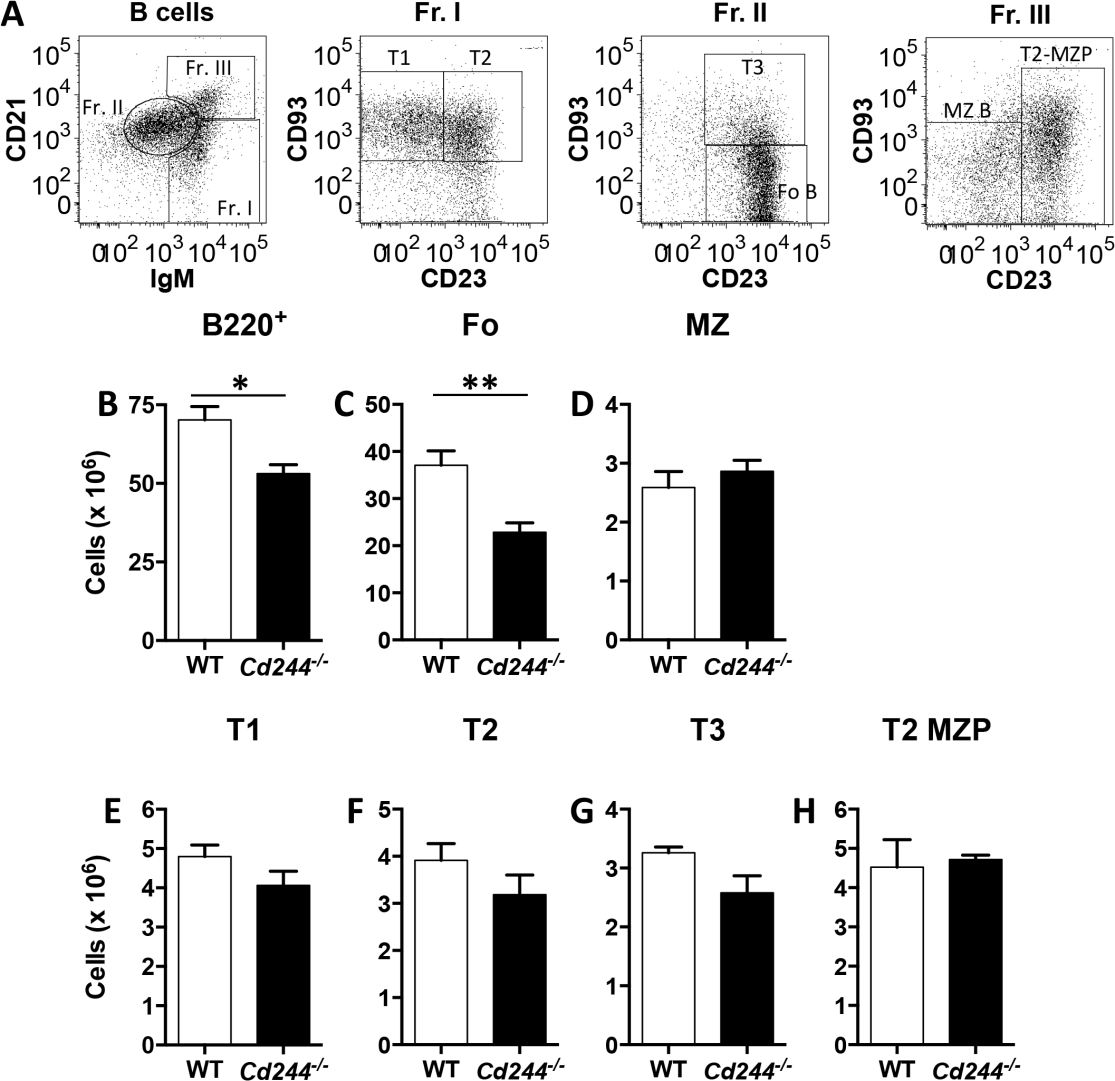
Total B cell numbers are reduced in the spleen of *Cd244* ^*-/-*^ mice due to a reduction in Fo B cells. A representative splenic B cell gating strategy is shown (A). The absolute number of B cells (B220^+^) (B) and that of the Fo (B220^+^IgM^int^CD21^int^CD23^+^CD93^-^) (C), MZ (B220^+^IgM^hi^CD21^hi^CD23^-^) (D), T1 (B220^+^IgM^hi^CD21^lo^CD23^-^CD93^+^) (E), T2 (B220^+^IgM^hi^CD21^lo^CD23^+^CD93^+^) (F), T3 (B220^+^IgM^int^CD21^int^CD23^+^CD93^+^) (G) and T2-MZP (B220^+^IgM^hi^CD21^hi^CD23^+^) (H) B cell subsets in the spleen of WT and *Cd244* ^*-/-*^ mice was determined by flow cytometry. Data are shown as the mean ± SE of four mice. *p<0.05; **p<0.01.

We determined whether the absence of 2B4 altered B cell differentiation in the spleen using a gating strategy to identify the Fo and MZ splenic mature B cells populations as well as their transitional (T) 1, T2 and T3 precursors (Fig 2A) [16]. We also analyzed the T2-MZ precursor (P), which is the immediate precursor to the MZ lineage (Fig 2A) [17]. When we quantitated total B cells, we found that the reduction in splenic cellularity in *Cd244* ^*-/-*^ mice was also due to a significant reduction in the number of B220^+^ B cells (Fig 2B). By quantitating the mature splenic B cell subsets we found that Fo (Fig 2C), but not MZ (Fig 2D), B cell numbers were significantly reduced. We next asked whether the reduction in Fo B cells was due to altered differentiation into the Fo lineage and found no difference in the transitional (T) 1 (Fig 2E) or T2 (Fig 2F) precursor populations between WT and *Cd244* ^*-/-*^ mice. We also found no difference in the T3 (Fig 2G) or T2-MZP (Fig 2F) subsets. These data indicate that the homeostasis of Fo B cells is uniquely altered by a global deletion in 2B4.

Collectively, our data demonstrate that neither development nor homeostasis of the myeloid lineage are regulated by 2B4. The data also demonstrate that even though 2B4 is highly expressed by NK cells [12], it also does not regulate their development or homeostasis. To our surprise, the reduced cellularity in the spleen of *Cd244* ^*-/-*^ mice was due to a reduction in both CD4^+^ T cells and Fo B cells. These data were unexpected because neither cell population is known to highly express 2B4 [4, 18, 19]. Thus their reduction in the *Cd244* ^*-/-*^ mouse is not likely to be cell intrinsic. Since 2B4 is the ligand for CD48, which is expressed by lymphocytes, it is more likely that a 2B4-expressing cell is interacting with and promoting CD4 and Fo B cell proliferation or survival via CD48. In this regard, ligation of CD48 on human B cells has been shown to provide an accessory signal for CD40 signaling including increasing B cell proliferation [20]. In addition, CD48 was also shown to augment both IgG and IgM production [20, 21]. For T cells, CD3 signaling in combination with CD48 ligation enhanced the phosphorylation level of the TCR ζ chain [22] and was found to enhance T cell proliferation in response to antigen and anti-CD3 [23, 24]. While the CD48^+^ cell involved in maintenance of CD4 and Fo B cell numbers is not known, it is possible that NK or iNK cells, which also express 2B4, play a role [2, 25–27].

### Splenic Fo B cells lack 2B4 expression, while MZ B cells express very low levels

**Fig 3:**
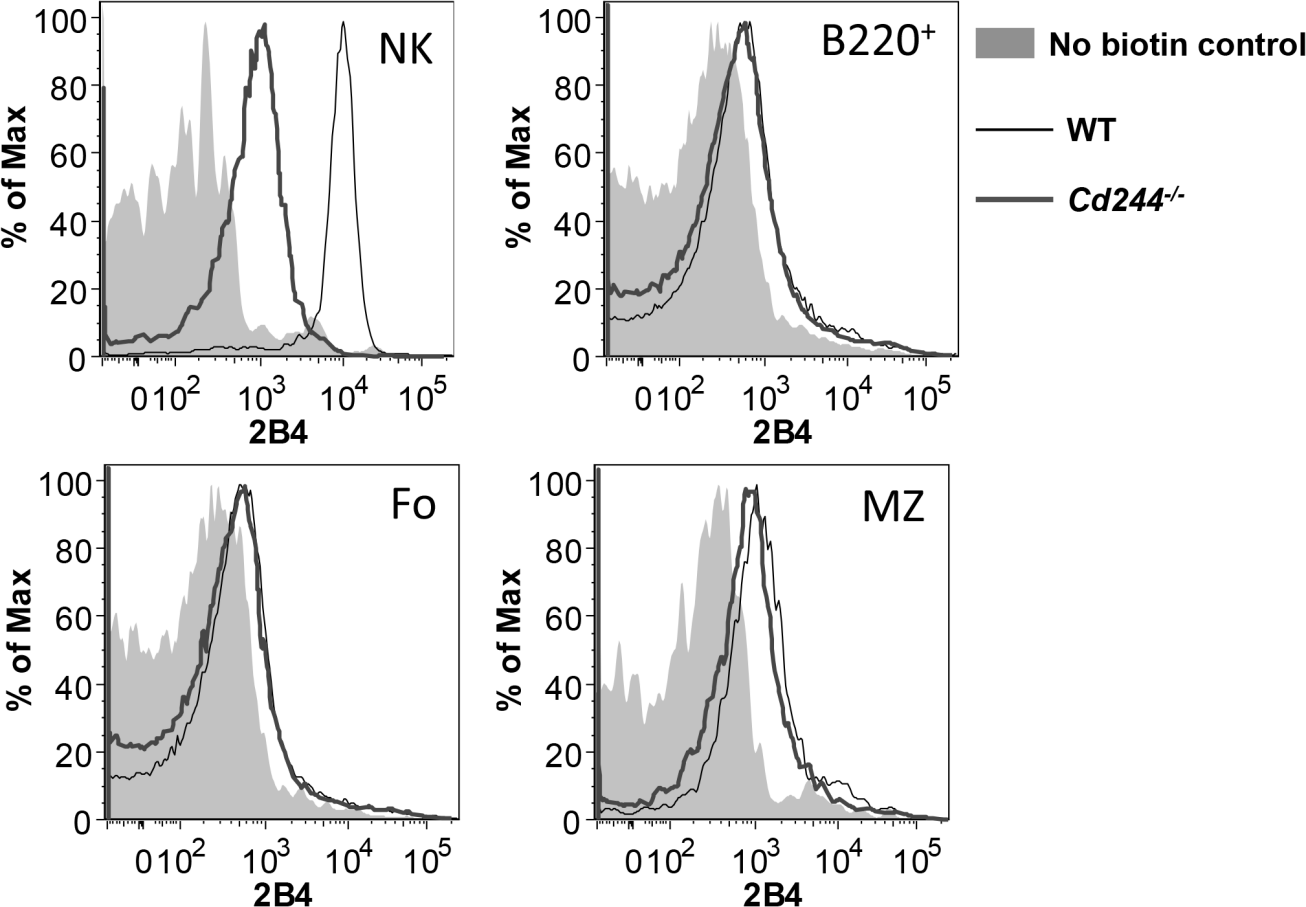
Splenic B cells express low levels of 2B4. Expression of 2B4 was evaluated on splenic NK cells (NK1.1^+^), B cells (B220^+^), Fo B cells and MZ B cells from WT and *Cd244* ^*-/-*^ mice. Histograms display overlaid staining profiles from the biotin negative control (shaded), WT mice (thin line) and *Cd244* ^*-/-*^ mice (thick line). Data shown are from one representative mouse of two.

We next determined whether splenic B cell subsets express 2B4 in the steady state by flow cytomtery. Because the lack of reported 2B4 expression by B cells could be due to a low expression level, we used a three step staining procedure to amplify the 2B4 signal. In addition, we performed the same staining in *Cd244* ^*-/-*^ mice to confirm specificity. As a positive control, we analyzed 2B4 expression on NK cells, which expressed 2B4 at a high level (Fig 3). 2B4 cell surface expression by flow cytometry was not evident on either the total B220^+^ or Fo subset (Fig 3). However, we found that MZ B cells express 2B4 at a very low level (Fig 3).

### 2B4 expression is dispensable for T-independent immune responses

**Fig 4:**
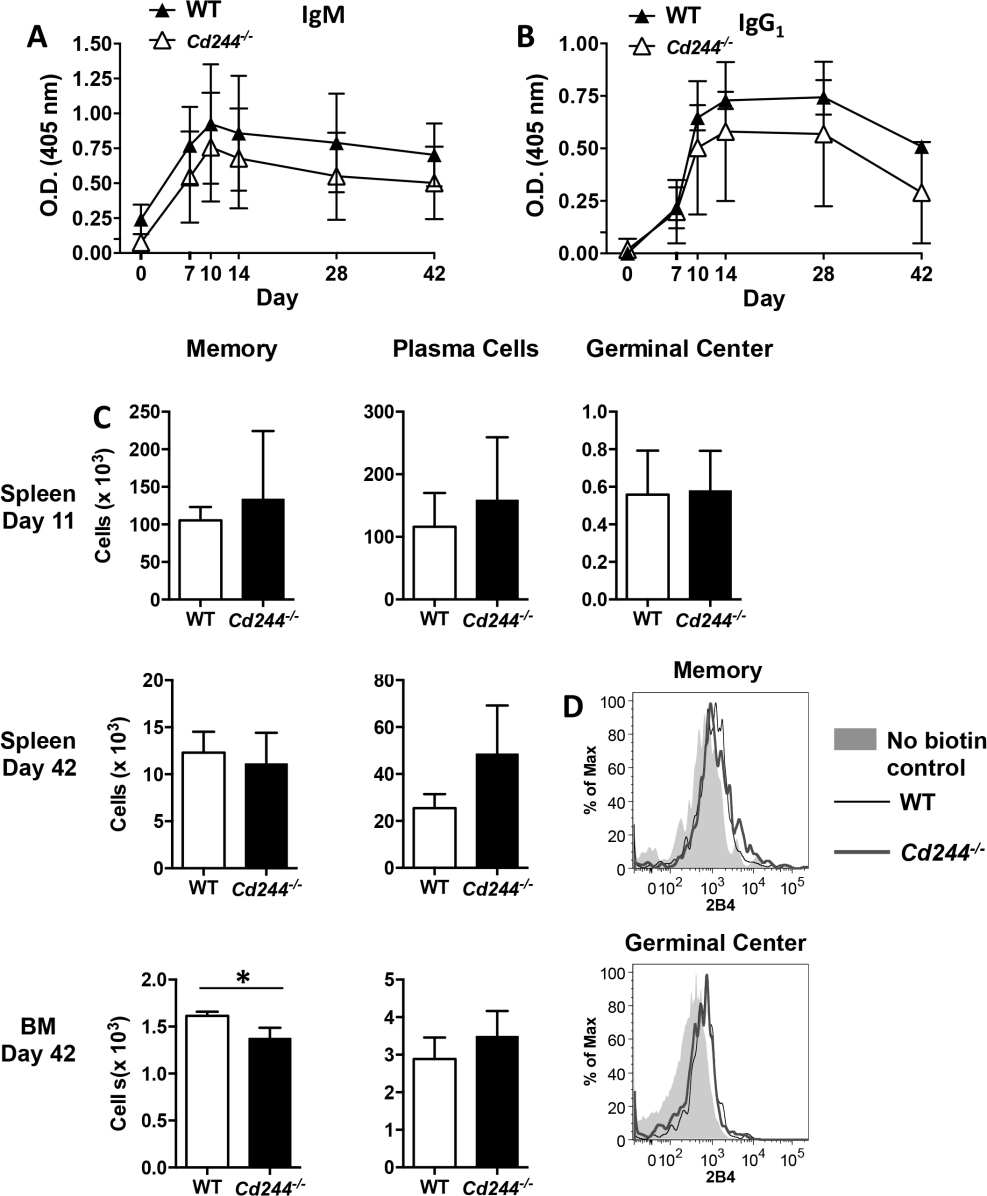
The T-dependent B cell response is unaltered in *Cd244* ^*-/-*^ mice. WT (closed symbol) and *Cd244* ^*-/-*^ (open symbol) mice were immunized (i.p.) with the T-dependent antigen NP-CGG (30 μg) that was alum precipitated. Blood was collected on days 0, 7, 10, 14, 28 and 42, and NP-specific IgM (A) and IgG_1_ (B) titers were determined by ELISA. The O.D. value for NP-specific IgM at the 1:800 dilution and NP-specific IgG_1_ at the 1:20,000 dilution are shown. Data shown are the mean ± SE of 8 mice for days 0 and 7; 7 mice for days 10, 14 and 28; and 4 mice for day 41. (C) The absolute number of memory (B220^+^IgD^-^NP(24)^+^CD38^+^), plasma cells (B220^+^IgD^-^IgM^-^IgG^+^NP(24)^+^CD138^+^) and germinal center (B220^+^GL-7^+^) B cells were determined in the spleen on days 11 (top panels) and 42 (middle panels) and in the BM on day 42 by flow cytometry. Data are shown as the mean ± SE of four mice. (D) Expression of 2B4 was evaluated on memory and germinal center B cell from WT and *Cd244* ^*-/-*^ mice 10 days after immunization. Histograms display overlaid staining profiles from the biotin negative control (shaded), WT mice (thin line) and *Cd244* ^*-/-*^ mice (thick line). Data shown are from one representative mouse of three. *p<0.05.

It was previously shown that SAP plays an essential role in T cell:B cell cognate interactions in the germinal center [28], indicating that SLAM molecules are involved in antibody production to T-dependent antigens. While a role for SLAM (CD150), was implicated in germinal center reactions [28], it is possible that other SLAM family members also play a regulatory role. To determine whether 2B4 regulates T-dependent antibody production, we immunized with NP-CGG and quantitated serum antigen-specific IgM and IgG_1_ on days 0, 7, 10, 14, 28 and 42. While there was a trend for WT B cells to produce higher levels of both IgM (Fig 4A) and IgG_1_ (Fig 4B), there was no significant difference as compared to *Cd244* ^*-/-*^ mice at any of the time points examined. When we quantitated memory B cells, we found no alteration in their number in the spleen on day 7 (data not shown) or on days 11 and 42 following immunization (Fig 4C). In contrast, BM memory cells were reduced on day 42 in *Cd244* ^*-/-*^ mice (Fig 4C). Consistent with similar levels of antigen-specific Ig, we found no difference in the number of plasma cells or germinal center B cells between WT and *Cd244* ^*-/-*^ mice at the timepoints examined in the spleen and BM (Fig 4C). Thus even though Fo B cells are reduced in *Cd244* ^*-/-*^ mice this only led to a minimal impact on the magnitude of the T-dependent antibody response. In addition, we did not observe 2B4 expression by splenic memory or germinal center B cells using flow cytometry on day 11 post-NP-CGG immunization (Fig 4D). The reduction in BM memory B cells is an interesting finding that could reflect a role for 2B4 in their migration from the spleen. In addition, 2B4 could be important in memory B cell maintenance specifically in the BM niche.

### *Cd244* ^*-/-*^ mice have increased numbers of peritoneal B1b cells and T-independent B cell responses

**Fig 5:**
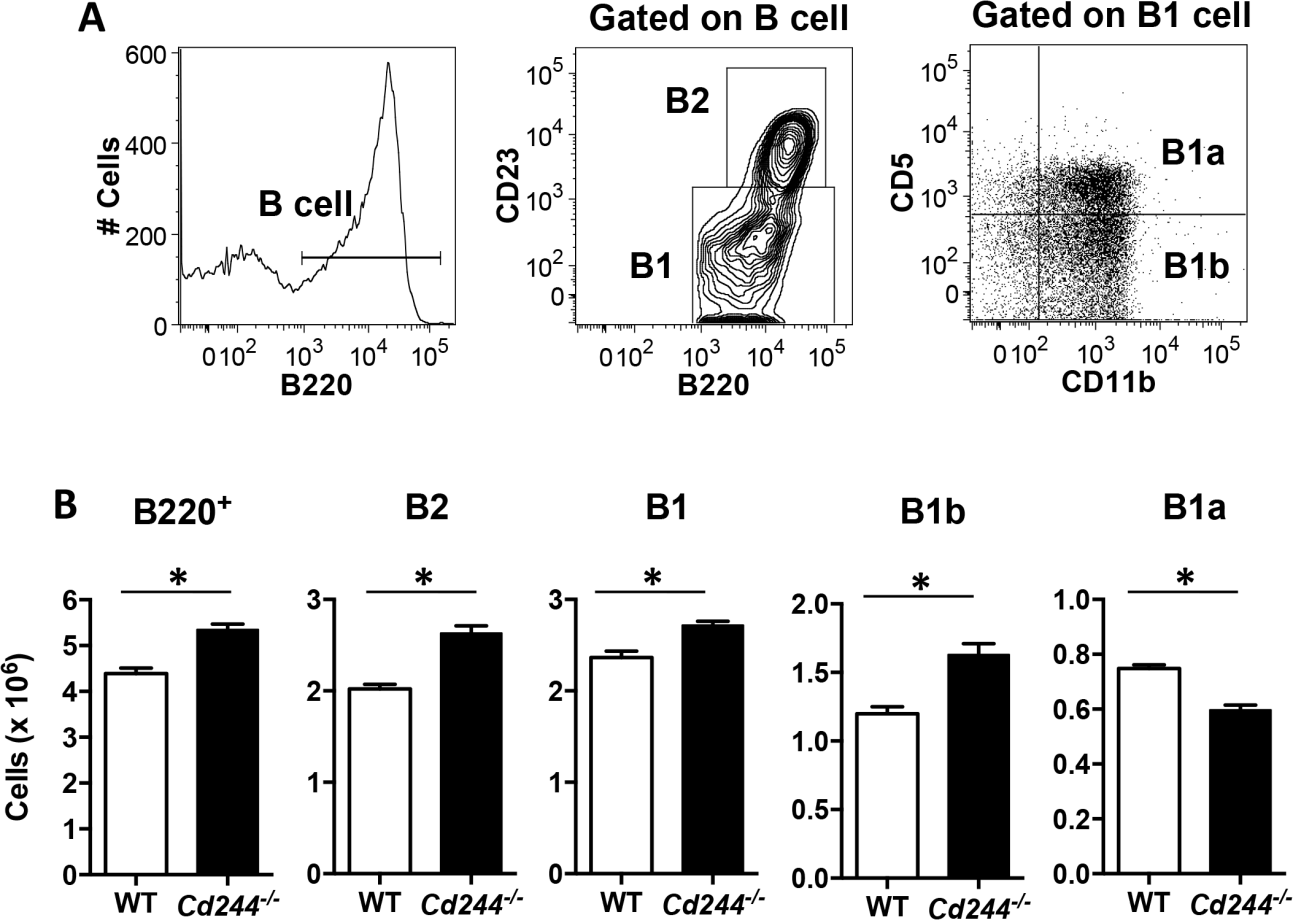
Total peritoneal cavity B cell numbers are elevated *Cd244* ^*-/-*^ mice due to an increase in B2 and B1b cells. (A) A representative peritoneal B cell gating strategy is shown. (B) The absolute number of B cells (B220^+^) and that of the B2 (B220^+^CD23^+^), B1 (B220^+^CD23^-^), B1b (B220^+^CD23^-^CD11b^+^CD5^-^) and B1a (B220^+^CD23^-^CD11b^+^CD5^+^) subsets in the peritoneal cavity of WT and *Cd244* ^*-/-*^ mice was determined by flow cytometry. Data are shown as the mean ± SE of four mice. *p<0.05.

Because T-independent immune responses were not altered due to a deficiency in 2B4, we next asked whether its loss impacted T-independent B cell responses. Because both MZ and peritoneal cavity B cells respond to T-independent antigens [17, 29], we first used a gating strategy to distinguish peritoneal cavity B cell subsets (Fig 5A) [14]. We found that total peritoneal cavity B cells (B220^+^) were increased in *Cd244* ^*-/-*^ mice as compared to WT (Fig 5B). This was due to a significant increase in both the conventional B2 and the B1 subsets (Fig 5B). When B1 cells were further differentiated, we found that while the B1b subset was significantly increased in *Cd244* ^*-/-*^ mice the reciprocal was true for B1a cells, which were significantly decreased (Fig 5B).

**Fig 6:**
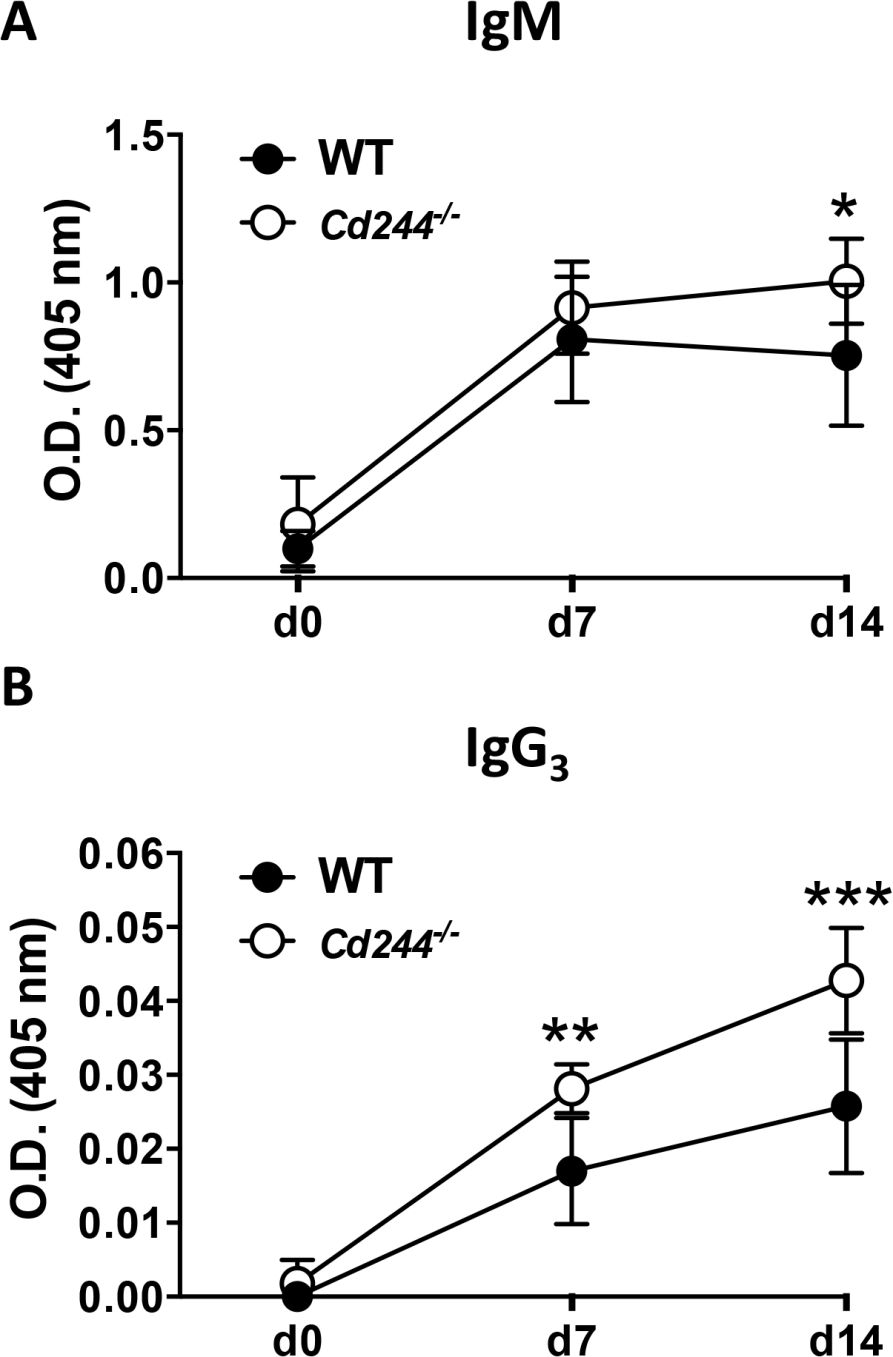
The IgM response to immunization with a T-independent antigen is enhanced in *Cd244* ^*-/-*^ mice. WT (closed symbol) and *Cd244* ^*-/-*^ (open symbol) mice were immunized (i.p.) with the T-independent antigen NP-ficoll (30 μg). Blood was collected on days 0, 7 and 14, and NP-specific IgM (top graph) and IgG_3_ (bottom graph) titers were determined by ELISA. The O.D. value for NP-specific IgM at the 1:800 dilution and NP-specific IgG_3_ at the 1:100 dilution are shown. Data shown are the mean ± SE of 8 mice. *p<0.05; **p<0.001; ***p<0.0001

Given the specific role for B1b cells in T-independent immune responses [30, 31], we next asked whether their increase in *Cd244* ^*-/-*^ mice would augment the B cell response to the T-independent antigen NP-ficoll. In T-independent immune responses, ligation of the BCR with a repeating antigen such as NP-ficoll induces BCR signaling that is sufficient to drive the production of antigen-specific Ig without the requirement for T cell help in the germinal center [32]. Following immunization with NP-ficoll, we measured serum NP-specific Ig on days 0, 7 and 14. As expected, levels of NP-specific IgM peaked on day 7 in WT mice that had begun to wane on day 14 (Fig 3A). In *Cd244* ^*-/-*^ mice, levels of NP-specific IgM were sustained on day 14 resulting in a significant increase in their serum levels (Fig 6A). Because T-independent immune responses lead to isotype class switching to IgG_3_, we also measured NP-specific IgG_3_ and found that it was significantly elevated on both day 7 and 14 in *Cd244* ^*-/-*^ mice (Fig 3B). Given that very little isotype-class switched IgG_3_ is generated following immunization with T-independent antigens the O.D. values are low. Even though the error bars are relatively small for the IgG_3_ data (Fig 6B), caution needs to be used to avoid overinterpretation of the data.

These cumulative data indicate that 2B4 expression by B cells is not modulating their ability to respond to T-independent antigens and does not regulate isotype class switching. However, 2B4 may play a role in restraining IgM and IgG_3_ production. Given that 2B4 can both positively and negatively regulate NK cell function depending upon whether SAP or EAT-2 are utilized, respectively, [3, 10], it is possible that 2B4 could also negatively regulate B cell effector functions. Given the low level of 2B4 expression by B cells, it is more likely that it regulates T-independent B cell responses in a cell extrinsic manner. However, we favor the hypothesis that the increase in antigen-specific Ig in *Cd244* ^*-/-*^ mice following immunization with a T-independent antigen is due to the increase in B1b cells, which are known to specifically be involved in such B cell immune responses [30, 31]. Whether 2B4 alters the development or homeostasis of B1b cells is not known.

In summary, we carefully phenotyped *Cd244* ^*-/-*^ mice for immune cell populations in the spleen and discovered that the reduced cellularity was due to a reduction in both CD4 T cells and Fo B cells. In contrast, increased numbers of peritoneal cavity B cells were present in *Cd244* ^*-/-*^ mice due to an increase in B2 and B1b cells. While 2B4 is an important regulator of NK cell function, when globally deficient, our study shows that it has minimal impact on T-dependent B cell immune responses. In contrast, the increase in B1b cells in *Cd244* ^*-/-*^ mice correlates with increased production of antigen-specific Ig in response to a T-independent antigen. The data also indicate that while both NK and iNK cells have been implicated in antibody production and isotype class switching [25, 26], this function is not likely mediated by 2B4:CD48 interactions.
